# Towards connecting biodiversity and geodiversity across scales with satellite remote sensing

**DOI:** 10.1111/geb.12887

**Published:** 2019-02-27

**Authors:** Phoebe L. Zarnetske, Quentin D. Read, Sydne Record, Keith D. Gaddis, Stephanie Pau, Martina L. Hobi, Sparkle L. Malone, Jennifer Costanza, Kyla M. Dahlin, Andrew M. Latimer, Adam M. Wilson, John M. Grady, Scott V. Ollinger, Andrew O. Finley

**Affiliations:** ^1^ Department of Forestry Michigan State University East Lansing Michigan; ^2^ Ecology, Evolutionary Biology, and Behavior Program Michigan State University East Lansing Michigan; ^3^ Department of Biology Bryn Mawr College Bryn Mawr Pennsylvania; ^4^ National Aeronautics and Space Administration Washington District of Columbia; ^5^ Department of Geography Florida State University Tallahassee Florida; ^6^ Swiss Federal Research Institute WSL Birmensdorf Switzerland; ^7^ SILVIS Lab, Department of Forest and Wildlife Ecology University of Wisconsin‐Madison Madison Wisconsin; ^8^ Department of Biological Sciences Florida International University Miami Florida; ^9^ Department of Forestry and Environmental Resources NC State University Research Triangle Park North Carolina; ^10^ Department of Geography, Environment, & Spatial Sciences Michigan State University East Lansing Michigan; ^11^ Department of Plant Sciences UC Davis Davis California; ^12^ Geography Department University at Buffalo Buffalo New York; ^13^ Department of Natural Resources and the Environment & Earth Systems Research Center University of New Hampshire Durham New Hampshire

**Keywords:** alpha diversity, beta diversity, biodiversity, elevation, gamma diversity, geodiversity, remote sensing, satellite, scale dependence, trees

## Abstract

**Issue:**

Geodiversity (i.e., the variation in Earth's abiotic processes and features) has strong effects on biodiversity patterns. However, major gaps remain in our understanding of how relationships between biodiversity and geodiversity vary over space and time. Biodiversity data are globally sparse and concentrated in particular regions. In contrast, many forms of geodiversity can be measured continuously across the globe with satellite remote sensing. Satellite remote sensing directly measures environmental variables with grain sizes as small as tens of metres and can therefore elucidate biodiversity–geodiversity relationships across scales.

**Evidence:**

We show how one important geodiversity variable, elevation, relates to alpha, beta and gamma taxonomic diversity of trees across spatial scales. We use elevation from NASA's Shuttle Radar Topography Mission (SRTM) and *c*. 16,000 Forest Inventory and Analysis plots to quantify spatial scaling relationships between biodiversity and geodiversity with generalized linear models (for alpha and gamma diversity) and beta regression (for beta diversity) across five spatial grains ranging from 5 to 100 km. We illustrate different relationships depending on the form of diversity; beta and gamma diversity show the strongest relationship with variation in elevation.

**Conclusion:**

With the onset of climate change, it is more important than ever to examine geodiversity for its potential to foster biodiversity. Widely available satellite remotely sensed geodiversity data offer an important and expanding suite of measurements for understanding and predicting changes in different forms of biodiversity across scales. Interdisciplinary research teams spanning biodiversity, geoscience and remote sensing are well poised to advance understanding of biodiversity–geodiversity relationships across scales and guide the conservation of nature.

## 1 INTRODUCTION

**Figure 1:**
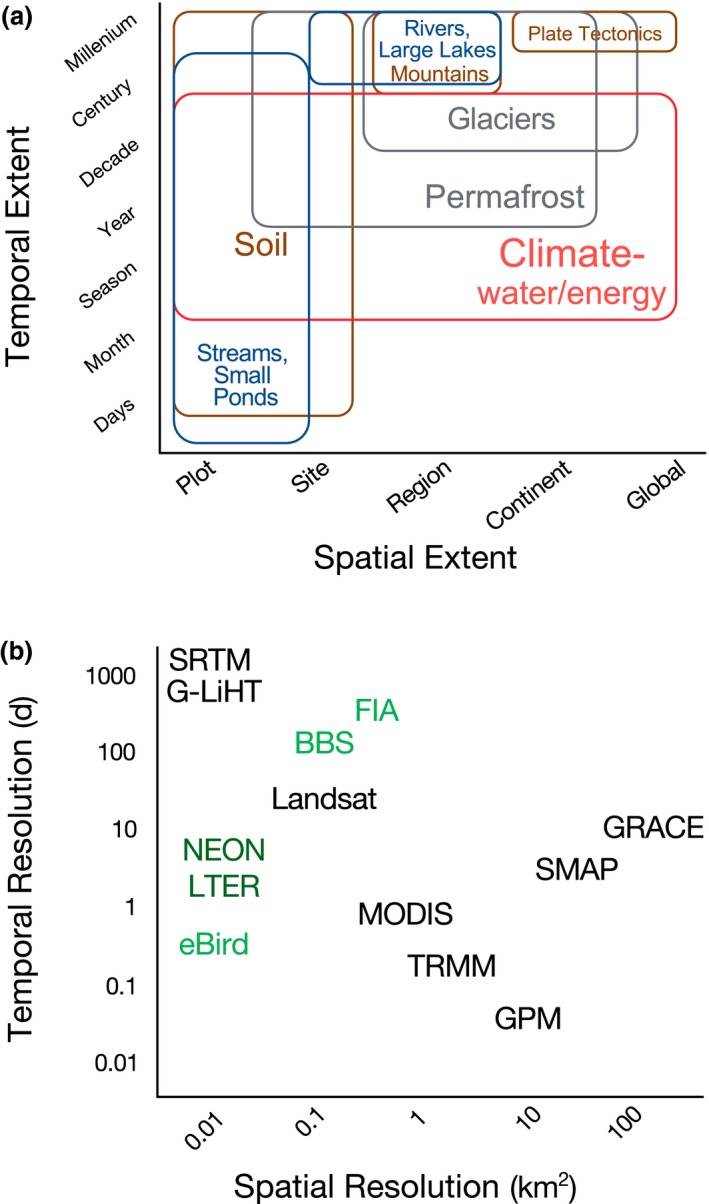
Geodiversity across scales. (a) Examples of geodiversity variables and the spatial and temporal extents at which they vary. Geodiversity encompasses abiotic components of the Earth's critical zone, specifically the lithosphere (brown), atmosphere (red), hydrosphere (blue) and cryosphere (grey) (Natural Resources Council, 2001; Parks & Mulligan, 2010). In general, surficial geodiversity at regional to global scales remains constant over short time‐frames (e.g., days to years), whereas local‐scale surficial geodiversity (e.g., micro‐topography and the physical and chemical properties of soil) vary over short to intermediate time‐frames (e.g., years to centuries). (b) Examples of satellite remotely sensed geodiversity (black). As point data, biodiversity data (green) are often high resolution, but are lacking in spatial and temporal extent. Networked sites, such as the National Ecological Observatory Network (NEON) and Long‐Term Ecological Research (LTER) sites, provide a combination of biodiversity and geodiversity (dark green). See an interactive table with a more complete list of NASA missions and products for geodiversity at: https://bioxgeo.github.io/bioXgeo_ProductsTable/, also in Suporting Information Appendix A. Additional abbreviations are as follows: BBS = Breeding Bird Survey; FIA = forest inventory and analysis; G‐LiHT = Goddard's LiDAR hyperspectral thermal imager; GPM = global precipitation measurement mission; GRACE = gravity recovery and climate experiment; MODIS = MODerate resolution imaging spectroradiometer; SMAP = soil moisture active passive; SRTM = shuttle radar topography mission; TRMM = tropical rainfall measuring mission [Colour figure can be viewed at wileyonlinelibrary.com]

The Earth is experiencing unprecedented global change, and species face uncertain fates. Global changes, including climate change, can cause species to shift their geographical ranges, resulting in the (dis)assembly of communities and novel or no‐analogue communities (Williams & Jackson, 2007) and ecosystems (Hobbs, Higgs, & Harris, 2009). Shifts in species ranges present logistical and ethical challenges for conservation prioritization (McLachlan, Hellmann, & Schwartz, 2007). In response, conservationists have proposed focusing on “geodiversity” as a means to preserve biodiversity, because areas with high geodiversity should harbour future biodiversity even with changing species composition (Gill et al., 2015; Lawler et al., 2015; Shaffer, 2015). This aptly named “conserving nature's stage” approach has been adopted by The Nature Conservancy to prioritize conservation of climate‐resilient sites (Beier & Brost, 2010; Shaffer, 2015). However, there are major knowledge gaps in our understanding and ability to predict how different forms of geodiversity influence biodiversity patterns across spatial and temporal scales (Figure 1a), and in adopting geodiversity data sources that span these scales (Figure 1b). Such knowledge is essential for effective conservation and policy, because many ecological processes and patterns are scale dependent (Levin, 1992; McGill, 2010).

Here, we present an approach to identify relationships between biodiversity and geodiversity across scales, provide results for a case study with alpha, beta and gamma tree diversity across a large region of the USA, and identify a suite of global and near‐global satellite remotely sensed geodiversity data sources spanning spatial and temporal scales.

## 2 FORMS OF GEODIVERSITY

A range of definitions of geodiversity exist; some include climate, whereas others explicitly exclude it (Gray, 2013; Lawler et al., 2015; Parks & Mulligan, 2010; Tukiainen, Bailey, Field, Kangas, & Hjort, 2017). In addition, geodiversity has commonly been treated categorically by thematically mapping climate, geology, geomorphology and soil features into land units (Anderson et al., 2015; Gray, 2013). To enable the use of continuous metrics in addition to ordinal and categorical ones, and to evaluate scaling relationships between biodiversity and geodiversity, we adopt the following definition of geodiversity: the set of abiotic processes and features of Earth's critical zone (lithosphere, atmosphere, hydrosphere and cryosphere). This comprehensive definition is inclusive of climate and reflects the fact that Earth's fluid and solid components have strong influences on each other (Jenny, 1994).

Like biodiversity, geodiversity can be described in different forms: as heterogeneity or variability within a site; as spatial turnover or the difference between sites; and as total variability across all sites. Unlike ground‐based biodiversity observations, geodiversity can be spatially continuous when measured via satellite remote sensing. Some forms of geodiversity are categorical (e.g., number of distinct features) and can be summarized with measures of diversity, whereas heterogeneity in continuous variables (e.g., elevation) can be determined using various metrics, such as standard deviation, kurtosis or texture measurements. Scaling relationships in geodiversity are common. For example, variation in soil moisture decreases with sampling extent (Choi, Jacobs, & Cosh, 2007), and the hydraulic geometry of stream channels (Leopold & Maddock, 1953) and river networks dictates how variability in slope changes with extent (Tarboton, Bras, & Rodriguez‐Iturbe, 1989).

Historically, it has been difficult to obtain reliable, consistent and continuous geodiversity data at regional or global scales. For this reason, spatial models of species distributions and biodiversity have traditionally used topographic data as a proxy variable for climatic or environmental variance, often combining them with gridded data interpolated from weather stations (Waltari, Schroeder, McDonald, Anderson, & Carnaval, 2014). However, recent work highlighted the wide range of methods and accuracies among products, showing that there is no “best” product and that higher‐resolution products are not necessarily more accurate (Behnke et al., 2016). Recent satellite missions, such as Landsat 8, Sentinel‐1, Sentinel‐2 and ICESat‐2, enable accurate and continuous acquisition of global geodiversity data in space and time (Figure 1b; Supporting Information Appendix A). The resulting data products include surface temperature, snow cover, clouds, topography and more. In addition, reanalysis products, such as MERRAclim (Vega, Pertierra, & Olalla‐Tárraga, 2017), combine satellite Earth observations (from 1979 to the present) to develop global models of geodiversity variables with coarse spatial resolution but high temporal resolution at temporally and spatially consistent scales. Although satellite‐derived estimates of temperature and rainfall have limitations (e.g., Maggioni, Meyers, & Robinson, 2016; Wan, Zhang, Zhang, & Li, 2004), their coverage is global or near global. For other geodiversity variables, such as soil moisture and groundwater (see Supporting Information Appendix A), no station‐derived global gridded products exist; thus, satellite remote sensing provides a needed data source. The gridded station dataset perhaps most widely used by ecologists is WorldClim (Hijmans, Cameron, Parra, Jones, & Jarvis, 2005). The newly released WorldClim‐2 dataset (Fick & Hijmans, 2017) now includes MODIS land surface temperature (LST) and cloud cover data, highlighting the importance of satellite remotely sensed data.

## 3 SATELLITE REMOTELY SENSED GEODIVERSITY DATA ARE CRUCIAL FOR UNDERSTANDING PATTERNS OF BIODIVERSITY

Geodiversity affects patterns of biodiversity directly and indirectly. Environmental conditions map directly to individuals’ physiological limits, whereas topographic complexity, habitat patch arrangement and geophysical feature configuration are associated with niche diversity. Physical barriers to movement and the persistence of landscape features can also affect biodiversity indirectly by enabling or restricting biotic interactions among species (Zarnetske et al., 2017) and affecting dispersal ability (Urban, Zarnetske, & Skelly, 2013). Components of geodiversity provide resources for species, including energy, water, nutrients and space (Parks & Mulligan, 2010).

Without satellite remotely sensed geodiversity data, it can be difficult to detect drivers of biodiversity patterns across large extents. With satellite remote sensing, spatially continuous, direct and independent measures of climate and elevation provide a means to identify when and where climate and elevation covary, enabling biodiversity scientists to ask persistent questions about the drivers of patterns of biodiversity at larger extents, with finer resolutions and at multiple scales.

## 4 KNOWLEDGE GAP: GEODIVERSITY AND BIODIVERSITY ACROSS SPATIAL SCALES

Despite their inherent coupling and individual scale dependence (Rahbek, 2005; Willig, Kaufman, & Stevens, 2003), biodiversity and geodiversity scaling relationships across taxa, regions and diversity measures are not well characterized. A recent study provides important insights into scaling relationships between the taxonomic alpha diversity of alien vascular plant species and the geodiversity of landforms from geological surveys and airborne remote sensing across Great Britain (Bailey, Boyd, Hjort, Lavers, & Field, 2017). In that study, landform diversity explained the most variation in alpha diversity at smaller spatial scales, whereas climate became more important at larger spatial scales. Yet biodiversity can be calculated in several forms: as alpha (within‐site), beta (turnover between sites, or the ratio of within‐site to across all sites) or gamma diversity (total across all sites). Further investigations could reveal how consistent biodiversity–geodiversity relationships are across species, regions and forms of biodiversity. Both the data and the computational tools are now becoming available to address these relationships (Supporting Information Appendix A). Here we ask: how do the relationships between geodiversity and different forms of biodiversity change across spatial scale? In Box 1 and associated Supporting Information, we present an approach to identify these biodiversity–geodiversity scaling relationships, illustrated with a case study of trees and elevation spanning 16.5° latitude in the western USA.

Globally, the highest levels of species richness are likely to be observed where high geodiversity, such as topographic heterogeneity, coincides with relatively productive and stable climatic regimes, such as the tropical Andes (Buckley & Jetz, 2008; Kreft & Jetz, 2007; Rahbek & Graves, 2001). One explanation for this pattern is that warmer, stable climates promote higher biodiversity (Hawkins, Porter, & Felizola Diniz‐Filho, 2003), and biodiversity promotes productivity and system sustainability (Tilman, Wedin, & Knops, 1996), even in fluctuating environments (Yachi & Loreau, 1999) and across heterogeneous landscapes (Oehri, Schmid, Schaepman‐Strub, & Niklaus, 2017). In addition, geodiverse regions, such as those that are tectonically active, exhibit high species richness and spatial turnover of species (Badgley et al., 2017). Such heterogeneous environments provide refuge habitat to support species persistence after environmental change and can isolate populations, resulting in speciation events (Stein, Gerstner, & Kreft, 2014). Increased richness in geodiverse areas may also occur because resource and habitat partitioning allow more species to coexist. Greater environmental heterogeneity at a given site is often correlated with higher species richness, but this relationship depends on the scale at which a species perceives the heterogeneity (Tews et al., 2004).

Although different species may exhibit different scaling relationships with geodiversity, these relationships are likely to be driven by common mechanisms at certain scales, regardless of taxonomic group. At continental to global scales, broad gradients of biological diversity result from interactions among climate, the degree of connectedness among populations and the amount of time over which evolutionary processes act (Forest et al., 2007). At these broad scales, beta diversity among sampling units should have a strong positive relationship with geodiversity because of differences in biogeographical and evolutionary histories (Barton et al., 2013). Regionally within a continent, variation in habitat complexity should influence biodiversity further. At regional scales, alpha and beta diversity should decline regardless of heterogeneity in geodiversity, because fewer new species are added from the regional species pool (Barton et al., 2013). At more local scales within an ecoregion, stochastic processes yield large variability in species occurrence among sites (Barton et al., 2013), resulting in increased variation in alpha and beta diversity. At these local scales, geodiversity is likely to interact with species’ life‐history characteristics, biotic interactions and dispersal to mediate species‐specific occurrences (McGill, 2010; Shmida & Wilson, 1985).

We expect the relationship between biodiversity and geodiversity to be stronger at broader extents where gamma diversity or macro‐scale richness is highest in both measures (MacArthur & Wilson, 1967; Rosenzweig, 1995; Turner, 1989). We expect that of all the forms of biodiversity, beta diversity will be linked most strongly with heterogeneity in geodiversity, because variation in geodiversity can lead to concomitant shifts in abiotic resource availability that alter habitat types and drive species turnover (Ricklefs, 1977). Biodiversity–geodiversity relationships are likely to be scale dependent owing to varying influences of local community assembly processes, such as dispersal limitation, biotic interactions and environmental filtering (e.g., Tello et al., 2015).

**BOX 1**

**Biodiversity–geodiversity scaling relationships in western U.S. trees**

We analysed if using american english spatial scaling relationships between geodiversity and different forms of tree biodiversity: alpha, beta and gamma. For geodiversity, we focused on variation in elevation because it is the most commonly used form of geodiversity (Stein et al., 2014), and many geodiversity variables are correlated with topography, especially at regional scales (Hjort & Luoto, 2012). We note that numerous geodiversity variables have been proposed (Gray, 2013; Parks & Mulligan, 2010), and investigation of their scaling relationships with different facets of diversity (taxonomic, functional and phylogenetic) is a needed area of research. Our approach provides a means to quantify such relationships. Data sources included western U.S. (CA, OR and WA) Forest Inventory and Analysis (FIA) plots, which consist of four 7.2 m fixed‐radius subplots in which all trees > 12.7 cm diameter at breast height are measured (Bechtold & Patterson, 2005), and a 1 arc s (*c*. 30 m) digital elevation model (DEM) from SRTM (NASA JPL, 2013; Supporting Information Appendix B).

To investigate biodiversity–geodiversity scaling relationships, we varied the grain size of analysis systematically. At different radii (5, 10, 20, 50 and 100 km) centred on each of the *c*. 16,000 FIA plots, we calculated tree taxonomic Shannon diversity (effective species number) and the standard deviation of all elevation pixels. We calculated the median abundance‐weighted effective species number (Jost, 2006) of all plots falling within the radius, including the focal plot (alpha), the mean abundance‐weighted pairwise dissimilarity of all pairs of plots in the radius, including the focal plot (beta), and the median abundance‐weighted effective species number of all plots in the radius as if they were a single community (gamma). We used the total basal area of each tree species in each plot as a measure of their abundance. We discarded all plots within 100 km of the political borders of the study region to avoid edge effects. To avoid pseudoreplication, we used an iterative search to generate a subsample of plots separated by ≥ 100 km, yielding *c*. 20 plots per subsample. We used generalized linear models (GLMs) for alpha and gamma diversity (gamma distribution and log link), and beta regression for beta diversity (Cribari‐Neto & Zeileis, 2010), to relate the univariate diversity of all the focal plots to the standard deviation of elevation. We assessed how standardized slope coefficients changed with spatial grain and computed confidence intervals by repeating the subsampling procedure 100,000 times (Box Figure 1).

**The effect of elevation variability on biodiversity varies with scale and form of diversity**

The relationship between topographic heterogeneity and tree gamma and beta diversity shows scale dependence, increasing in magnitude between 5 and 20 km, then plateauing (Box Figure 1d). Overall, tree gamma diversity is most strongly related to topographic heterogeneity (Box Figure 1c; Supporting Information Appendix B). The maximal magnitude of the biodiversity–geodiversity relationship at intermediate to large grain sizes might be attributable, in part, to tree biodiversity levelling off at larger grain sizes (50–100 km), whereas elevational variability increases monotonically with scale (Box Figure 1a–d). This pattern suggests that for a given extent, there is a maximal grain size where the biodiversity–geodiversity relationship is strongest. The form of this relationship is likely to be related to historical processes or biogeography involving topographic constraints that affect dispersal (e.g., at treeline, across large rivers or at biome boundaries). For example, particular tree species may thrive on steep slopes, whereas other species are found in flat regions or riparian zones, but this sorting is unrelated to how many species are present in these different habitats. At even larger spatial extents, such as continents or the globe, we expect that the biodiversity–geodiversity relationship will weaken as historical processes at the biome scale play a larger role in determining patterns of biodiversity.

**Box Figure 1:**
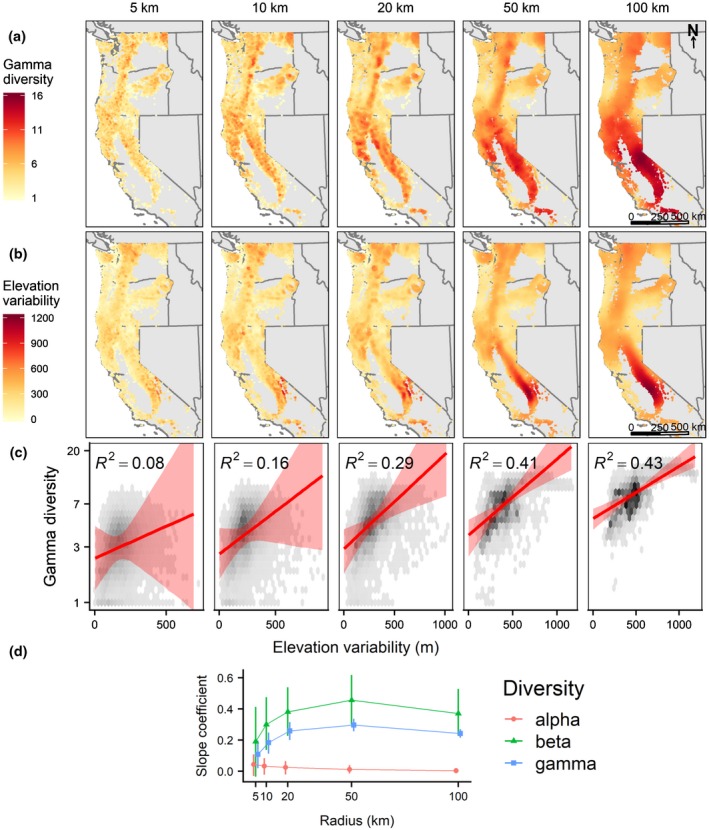
Patterns of variation in tree biodiversity and topographic geodiversity depend on the scale at which they are measured or summarized. For the analysis, total extent remained constant (CA, OR and WA, USA), and grain size (radius encompassing data) varied. Locations depicted in maps are fuzzed FIA coordinates (Woudenberg et al., 2010). (a) Forest inventory and analysis (FIA) tree taxonomic gamma diversity at 5–100 km. (b) Standard deviation of elevation at 5–100 km. (c) The relationship between gamma diversity and elevation variability (*SD* of elevation), the median *R* ^2^ value of the models, and the shaded red band bounded by the 2.5th and 97.5th percentiles of the predicted values from the models. (d) Scaling relationships between variation in biodiversity and geodiversity, represented as the standardized slope coefficients from generalized linear models (GLMs) for alpha and gamma diversity, and beta regression models for beta diversity for each scatter plot in panel (c) above versus distance (in kilometres; grain size); error bars represent 25th–75th percentiles, and points are offset slightly to avoid overlap. Standardized slopes are the increase in number of standard deviations in diversity with 1 m increase in the standard deviation of elevation. See the Supporting Information (Appendix B) for alpha‐ and beta‐diversity maps and relationships. Values of gamma diversity for each combination of point and radius are the total aggregated diversity value of all plots within the radius centred at the point [Colour figure can be viewed at wileyonlinelibrary.com]

## 5 WAYS FORWARD

### 5.1 The future of geodiversity with satellite remote sensing

Satellite remote sensing elucidates biodiversity–geodiversity scaling relationships because data are continuously measured and can be aggregated across different extents and grains. The field of remote sensing is changing rapidly, with advances in computational and engineering allowing researchers to measure geodiversity, capture climate variability and map biodiversity patterns at multiple scales. Advances include new satellite missions that measure geodiversity, publicly available big data from online biodiversity repositories, and new statistical approaches to model abiotic and biotic drivers of multiple species distributions simultaneously. Satellite missions provide global or near‐global data coverage for generating geodiversity variables at increasingly fine spatial resolutions and to help address scaling questions (Supporting Information Appendix A). For example, with the combination of the SRTM and ASTER global DEMs, it is possible to calculate a variety of topographic diversity variables at 30 m resolution at a near‐global extent (Simard, Neumann, & Buckley, 2016). The rise of RADAR and LiDAR technology on air‐ and spaceborne platforms makes it possible to quantify fine‐scale topographic geodiversity (e.g., Parks & Mulligan, 2010). Climatic variables can be derived from MODIS (e.g., Wan et al., 2004), SMAP (e.g., Chan et al., 2018), GPM (e.g., Hou et al., 2014), AMSR (e.g., Parinussa, Holmes, Wanders, Dorigo, & Jeu, 2015) and other spaceborne sensors and platforms, and provide the basis for compiling standard bioclimatic variables at multiple spatial and temporal scales. Other satellite sensors, such as GRACE and ICESat‐2, can provide new information about groundwater and the cryosphere, respectively (e.g., Kwok, 2018; Landerer & Swenson, 2012). These advances are coupled with a long history of optical satellite and airborne data. When coupled with multispectral (e.g., Landsat, MODIS, VIIRS and AVHRR) and hyperspectral (e.g., Hyperion and proposed future missions) capability, these data enable measures of geodiversity (soil cover and rock type) and biodiversity (ecosystem types, plant communities, functional types, species identities and genetic variability).

### 5.2 Challenges for data integration

Scale mismatches and gaps in measurements may hinder the integration of disparate datasets (Anderson, 2018). Biodiversity measurements tend to be measured at single locations or in small plots, whereas remotely sensed geodiversity variables are generally at least an order of magnitude larger (Figure 1b). Remotely sensed geodiversity measurements are more likely to be global and repeated through time, yet biodiversity observations remain relatively sparse geographically and phylogenetically and are rarely repeated through time (Amano, Lamming, & Sutherland, 2016; Urban et al., 2016). Furthermore, the spatial and temporal resolutions of different geodiversity datasets often do not match (Figure 1b), making it necessary to model or resample variables. In general, the time‐scales over which biodiversity changes are likely to be shorter than those over which most geodiversity changes. However, both forms of diversity can change over short to long time‐scales. Geodiversity in fluvial systems can change markedly within minutes to decades or more, whereas orogenic events often span millennia (Figure 1a). Biodiversity at a given location can change rapidly (minutes to decades), as a result of habitat destruction or species invasion, or gradually (centuries to millennia), owing to evolution.

The use of remotely sensed metrics of geodiversity to predict biodiversity at certain scales will require knowledge of the scales and processes by which geodiversity drives biodiversity for different taxonomic groups and life‐history characteristics. Multivariate or ensemble geodiversity measures (Parks & Mulligan, 2010) should be interpreted carefully, because their aggregate nature is likely to mask important biodiversity–geodiversity relationships. Although exploratory research and data mining will help to identify key metrics and scales, more process knowledge is necessary to pair specific types of biological responses with geodiversity drivers at specific scales. Feedbacks among geodiversity drivers at multiple scales are likely to exist; therefore, understanding cross‐scale interactions (Soranno et al., 2014) is a research priority.

Finally, although satellite remotely sensed data are often publicly available, the need to use big data management (Kelling et al., 2009) and remote sensing techniques can be a hurdle for investigators. Although many ecologists are familiar with MODIS and Landsat data products, they may not be aware of other products, such as GRACE, SMAP or Hyperion. Such underused geodiversity measures should be assessed for their ability to explain and predict biodiversity. The rise of cloud‐based computing platforms, such as Google Earth Engine, can facilitate data accessibility and operability.

### 5.3 Networks and interdisciplinary research opportunities

Coordinated observation networks and interdisciplinary research teams are well positioned to advance knowledge of biodiversity–geodiversity linkages across scales and, ultimately, to improve forecasts of future biodiversity change. Observation networks, such as the National Ecological Observatory Network (NEON; Keller, Schimel, Hargrove, & Hoffman, 2008), provide a means to scale up ecology and can be used to investigate biodiversity–geodiversity relationships using co‐located ground‐based biodiversity observations and remotely sensed geodiversity from tower‐based, airborne and satellite platforms. Teams of researchers and practitioners that span disciplines can more effectively address fundamental and applied questions that are essential to forecast changes to biodiversity across scales (Heffernan et al., 2014; Pettorelli, Safi, & Turner, 2014; Reinhardt, Jerolmack, Cardinale, Vanacker, & Wright, 2010). In this age of big data, the combination of coordinated research networks and interdisciplinary teams of investigators may be the best way forward to advance the conservation of nature.

## ACKNOWLEDGEMENTS

Funding for the bioXgeo working group was provided by the National Aeronautics and Space Administration (NASA) Ecological Forecasting Program, Earth Science Division, Grant #NNX16AQ44G awarded to PLZ, KMD, and SR. Additional support for PLZ, KMD, QDR, JMG, and AOF came from Michigan State University, and for PLZ: USDA NIFA Hatch Project 1010055. Additional support for SR came from Bryn Mawr College. AOF was also supported by National Science Foundation (NSF) grants DMS‐1513481, EF‐1137309, EF‐1241874, EF‐1253225. Additional support for AMW came from NASA Grant #NNX16AQ45G. SVO was also supported by NSF grants 1638688, 1237491, 1637685, and USDA grant #NH00634. KDG was supported by a AAAS Science & Technology Policy Fellowship served at NASA. We thank Woody Turner, the editors, and two anonymous reviewers, for insightful comments. We thank the National Center for Ecological Analysis and Synthesis (NCEAS) for hosting working group meetings. The views expressed in this paper do not necessarily reflect those of NASA, the United States Government, or the American Association for the Advancement of Science. Forest Inventory and Analysis location data were provided through the Forest Service Agreement No. 17‐MU‐11261919‐021.

## BIOSKETCH

The authors are members of a NASA Biodiversity working group, “bioXgeo”. The goal of the working group is to connect biodiversity and geodiversity with remote sensing across scales in order to advance predictive models of biodiversity in an era of rapid global change. Collectively, they have expertise in global change ecology, biodiversity, community ecology, geodiversity, species distribution modeling, remote sensing, and spatial statistics. The bioXgeo github site is available at: https://github.com/bioxgeo.

## Data Availability

Tree and location data used to generate these analyses cannot be published, according to Forest Service Agreement no. 17‐MU‐11261919‐021. Digital elevation model data from the NASA Shuttle Radar Topography Mission are freely available from the United States Geological Survey (https://lpdaac.usgs.gov/dataset_discovery/measures/measures_products_table/srtmgl1_v003).
