# Supplementary figures and images for: Towards connecting biodiversity and geodiversity across scales with satellite remote sensing
Source: Glob Ecol Biogeogr. 2019 Feb 27;28(5):548–56. doi: 10.1111/geb.12887 (PMC6559161; doi:10.1111/geb.12887)

Alpha-  
diversity

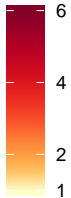

5 km

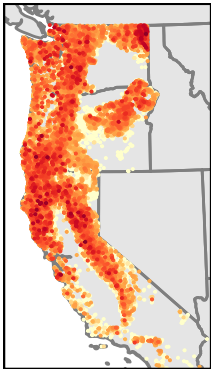

10 km

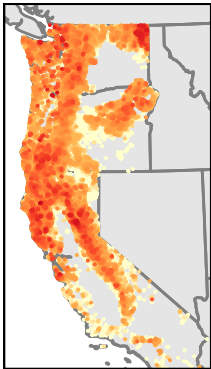

20 km

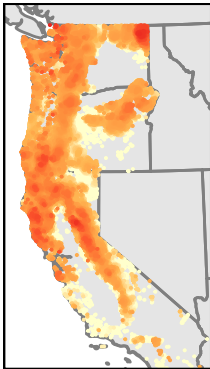

50 km

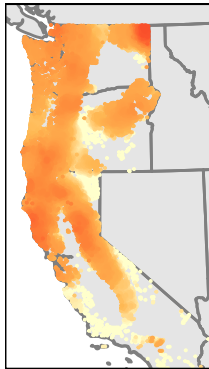

100 km

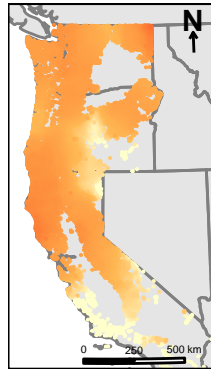

Supplement: Supplementary file 1 [file GEB-28-548-s001.pdf]

Beta-  
diversity

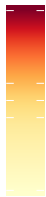

5 km

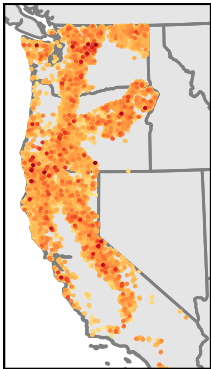

10 km

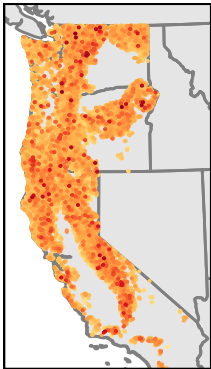

20 km

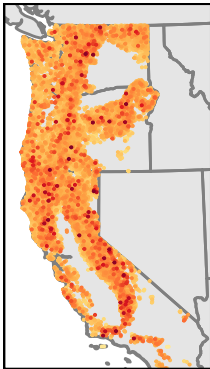

50 km

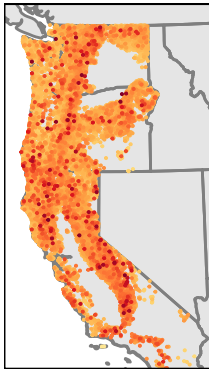

100 km

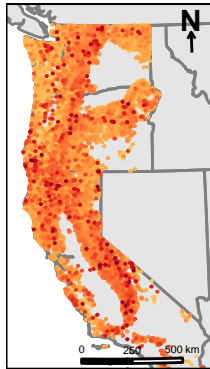

Supplement: Supplementary file 2 [file GEB-28-548-s002.pdf]

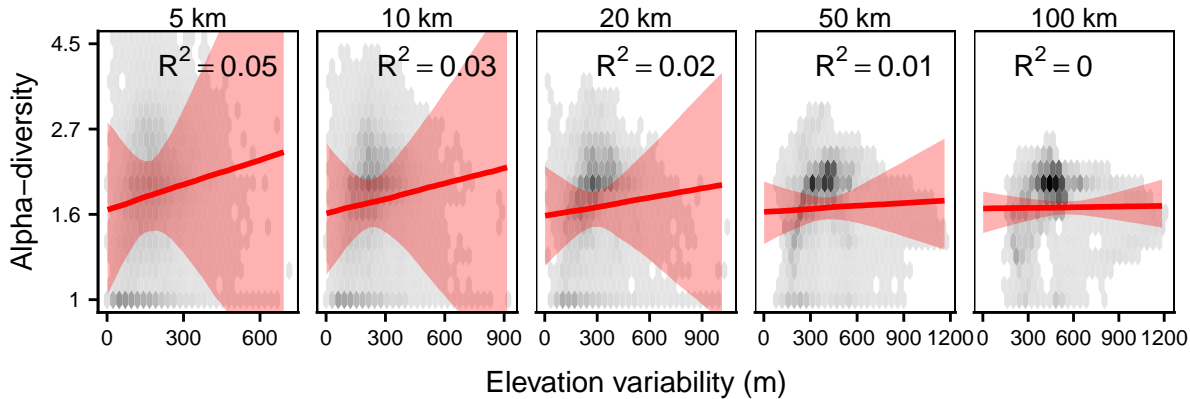

Supplement: Supplementary file 3 [file GEB-28-548-s003.pdf]

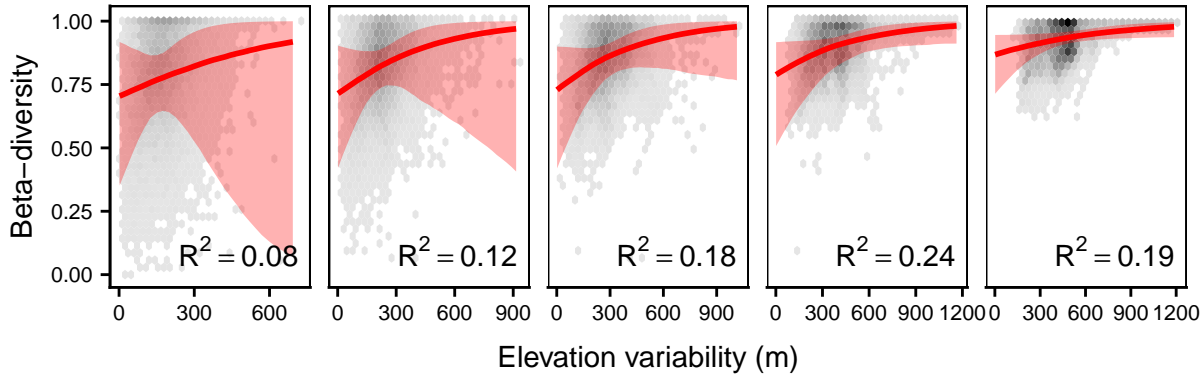

Supplement: Supplementary file 4 [file GEB-28-548-s004.pdf]

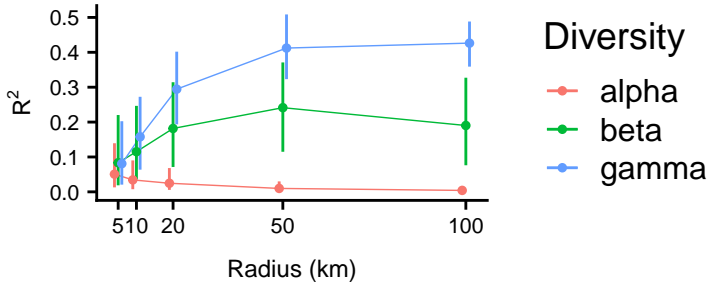

Supplement: Supplementary file 5 [file GEB-28-548-s005.pdf]
