# Supplementary material for: Towards connecting biodiversity and geodiversity across scales with satellite remote sensing
Source: Glob Ecol Biogeogr. 2019 Feb 27;28(5):548–56. doi: 10.1111/geb.12887 (PMC6559161; doi:10.1111/geb.12887)
Supplement: Supplementary file 6 [file GEB-28-548-s006.docx]

### Supplemental Materials List (GEB_12887)

Appendix A. Table of geophysical remote sensing products from NASA and their associated geodiversity variables.

Appendix B. Calculation of taxonomic diversity of FIA tree communities.

Appendix B-1. Preparation of Forest Inventory and Analysis dataset.

Appendix B-2. Diversity calculations.

Appendix B-2.1. Alpha and gamma diversity.

Appendix B-2.2. Beta diversity.

Appendix B-3. Additional figures related to Box 1.

Supplemental Figure B-1. Alpha diversity of trees in FIA plots in the Pacific Northwest region.

Supplemental Figure B-2. Beta diversity of trees in FIA plots in the Pacific Northwest region.

Supplemental Figure B-3. Generalized linear models with gamma distribution and log-link of alpha diversity versus the standard deviation of elevation.

Supplemental Figure B-4. Beta regressions of beta diversity versus the standard deviation of elevation.

Supplemental Figure B-5. R^2^ values from biodiversity-geodiversity relationships shown in Supplemental Figures B-3 and B-4, and Box Fig. 1c.

### APPENDICES

### APPENDIX A

Table 1. Available geophysical remote sensing products from NASA which provide geodiversity variables, with their spatial and temporal scales noted.

Online interactive table available at: [https://bioxgeo.github.io/bioXgeo_ProductsTable/](https://urldefense.proofpoint.com/v2/url?u=https-3A__bioxgeo.github.io_bioXgeo-5FProductsTable_&d=DwMFaQ&c=nE__W8dFE-shTxStwXtp0A&r=ci9qYLRC4ycgiVh0fkGRBA&m=fF4L6aO6-3eazpk2_wKX8Pm8n1r2DsodkxFOsXTxnm4&s=R7cDRIfhevscw2yO4YYNvGfal_rZ0XPiU1kb0Nv0Wm4&e=)

###

### APPENDIX B: Calculation of taxonomic diversity of FIA tree communities

#### B-1. Preparation of Forest Inventory and Analysis dataset

We obtained USFS Forest Inventory and Analysis (FIA) data, including measurements and locations, for the Pacific Northwest region (California, Oregon, Washington, USA) (Forest Service Agreement No. 17-MU-11261919-021). Forest plots surveyed according to the FIA protocol consist of four subplots, each circled with 7.3 m radius, located 36.6 m from one another in a three-pointed star pattern. See Bechtold *et al.* (2005) and https://www.fia.fs.fed.us/library/field-guides-methods-proc/docs/2016/core_ver7-1_10_2016-opt.pdf for more detailed description of the survey protocols. We retained only plots identified as natural forest by excluding plots with no trees and plots identified as plantation forests, resulting in approximately 16,000 plots across the three states. Each tree in each subplot is identified to species, and its diameter at breast height is recorded. Using the diameters to calculate basal area of each individual tree, we summed the basal areas within each species to estimate the relative abundance of each species in each subplot. Any discrepancies in species names were resolved to the most recent taxonomy.

#### B-2. Diversity calculations

We calculated abundance (basal area)-weighted diversity metrics for species present at each plot. We used the most recent survey as a single time point for each plot. Our decision to use basal area as a surrogate for tree abundance is consistent with many previous studies that computed diversity metrics for tree communities (e.g., Risser & Rice, 1971; Liang *et al.*, 2007; Grossiord *et al.*, 2014)

We calculated alpha, beta, and gamma diversity at a number of different radii around each FIA plot by taking the median diversity of all plots in the radius, including the focal plot (alpha), the mean pairwise Sørensen dissimilarity of all pairs of plots in the radius, including the focal plot (beta), and the aggregated diversity of all plots in the radius as if they were a single community (gamma). We calculated the mean arcsine-square root transformed value in the case of beta diversity, then back-transformed to the original scale (0 to 1). The radii for which we calculated diversities included 5,10, 20, 50, and 100 km; a subset of these results are presented in the manuscript.

##### B-2.1 Alpha and gamma diversity

We calculated taxonomic alpha diversity (Shannon diversity of a local community) for FIA tree communities. We calculated diversity indices for communities aggregated at the plot level (aggregating the four subplots making up one plot). For each plot and radius, we calculated alpha diversity within that radius by taking the median diversity value for all plots or routes (including the focal plot) located inside the circle defined by the radius around the focal plot. For gamma diversity, the diversity of a region that consists of multiple local communities, we aggregated all the plots within the focal circle to a single community, and calculated taxonomic, functional, and phylogenetic diversity of that community. We calculated basal-area-weighted Shannon alpha and gamma diversity as follows: $H' = \sum_{i=1}^{R} -p_{i} ln p_{i}$ , where R is species richness and p_i_ is the basal area of species i. We expressed this as true diversity, or effective species number, with q = 1 by exponentiating Shannon diversity (Jost, 2007).

##### B-2.2 Beta diversity

We calculated taxonomic beta diversity (turnover of diversity among local communities) for FIA tree communities. Beta diversity is defined as the variation in community composition across multiple local communities. To determine beta diversity at a point, it is necessary to define the kernel or radius within which variation in community composition is taken into account. For the FIA dataset, we aggregated species abundances of each plot and calculated beta diversity for each plot at a number of different radii around the focal plot; as the radius increases, the number of pairwise comparisons among plots also increases as more plots fall within the kernel.

We calculated beta diversity with the pairwise dissimilarity method using the vegdist() function from the R package vegan (Oksanen *et al.*, 2018) and taking the mean of the pairwise Sørensen dissimilarity (transformed with the arcsine-square root transform, then back-transformed to the original scale, 0-1) of all local communities within a particular radius of the focal plot.

#### B-3. Additional figures related to Box 1.


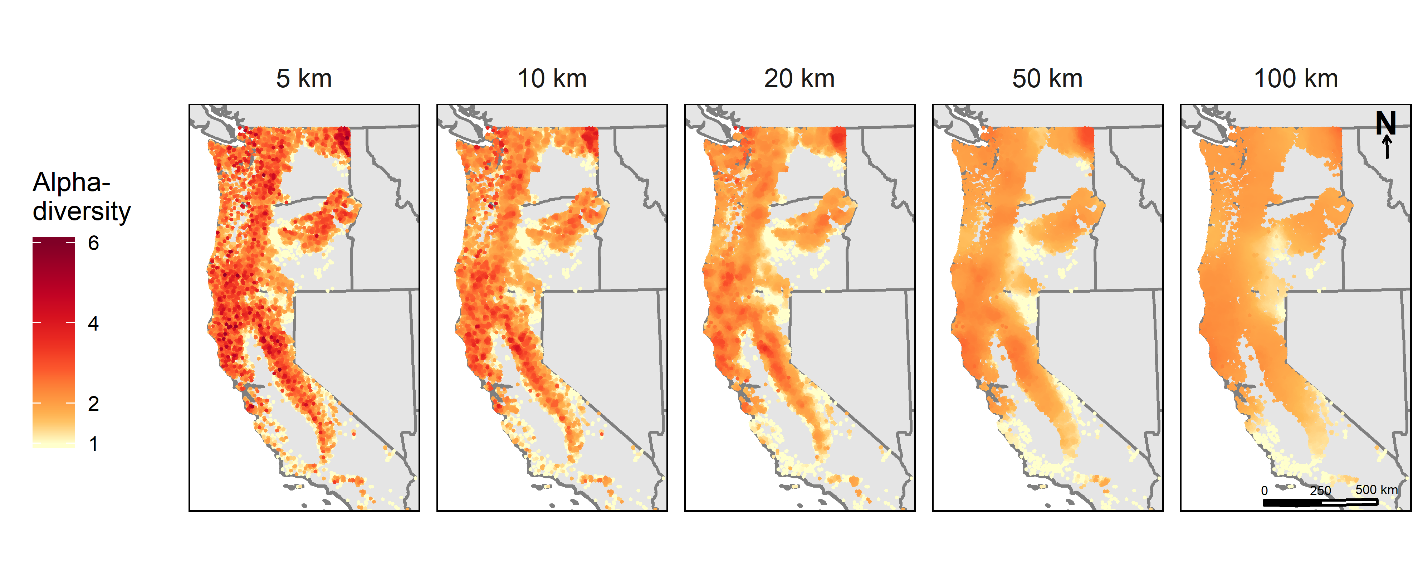


Supplemental Figure B-1. Alpha diversity of trees in FIA plots in the Pacific Northwest region, expressed as effective species number with q=1, or the exponential of Shannon diversity. Alpha diversity values for each combination of point and radius are the median diversity value of all plots within the radius centered at the point.


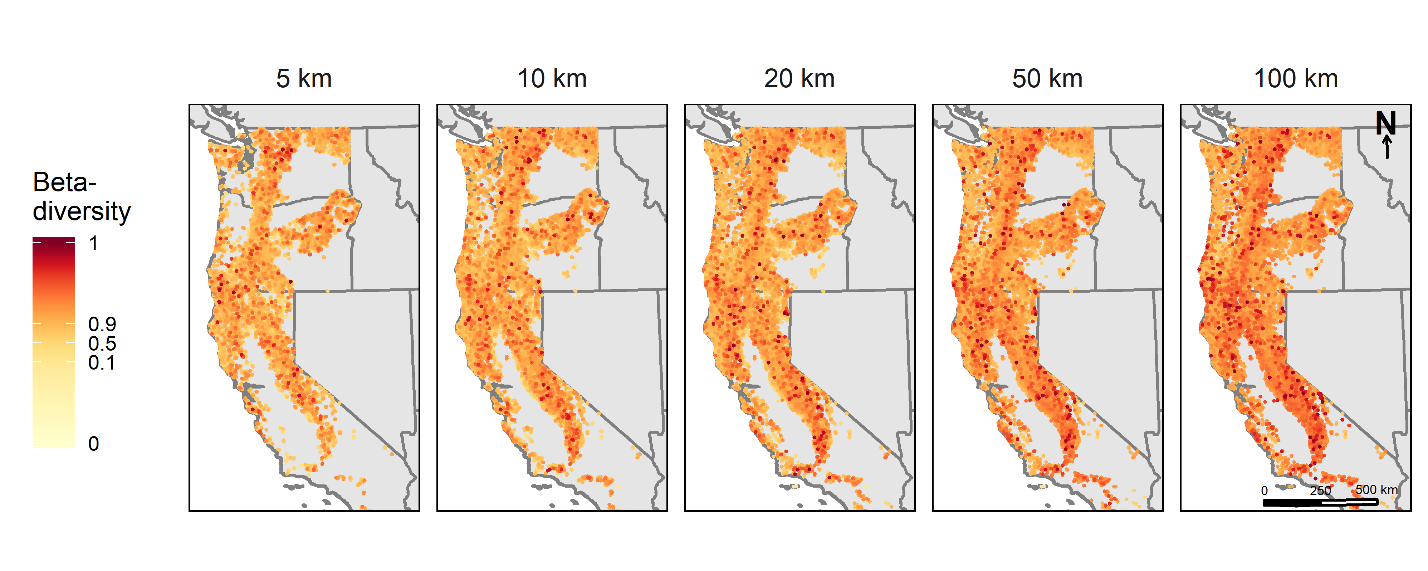


Supplemental Figure B-2. Beta diversity of trees in FIA plots in the Pacific Northwest region, expressed as effective species number with q=1, or the exponential of Shannon diversity. Beta diversity of the regions around the plots are depicted in logit scale to improve the distinction between values at the higher end of the scale near 1.


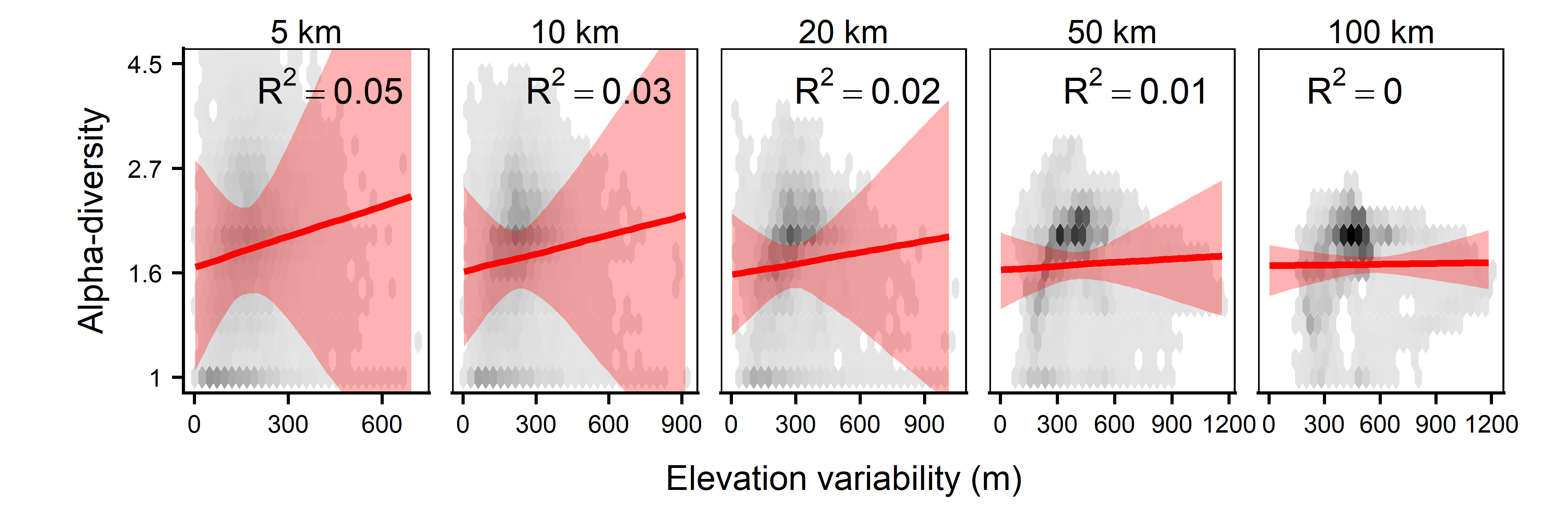


Supplemental Figure B-3. Generalized linear models with gamma distribution and log-link of alpha diversity (Shannon diversity) versus the standard deviation of elevation. Density of points in the scatterplot is represented by shading of hexagonal areas to avoid overplotting. The dark red line is the median predicted value of models fit with 100,000 spatially stratified random subsamples of the full dataset, each with approximately n=20. The shaded red area is bounded by the 2.5% and 97.5% percentiles of the predicted values from the regressions. The median R^2^ value of the models is shown in each panel.


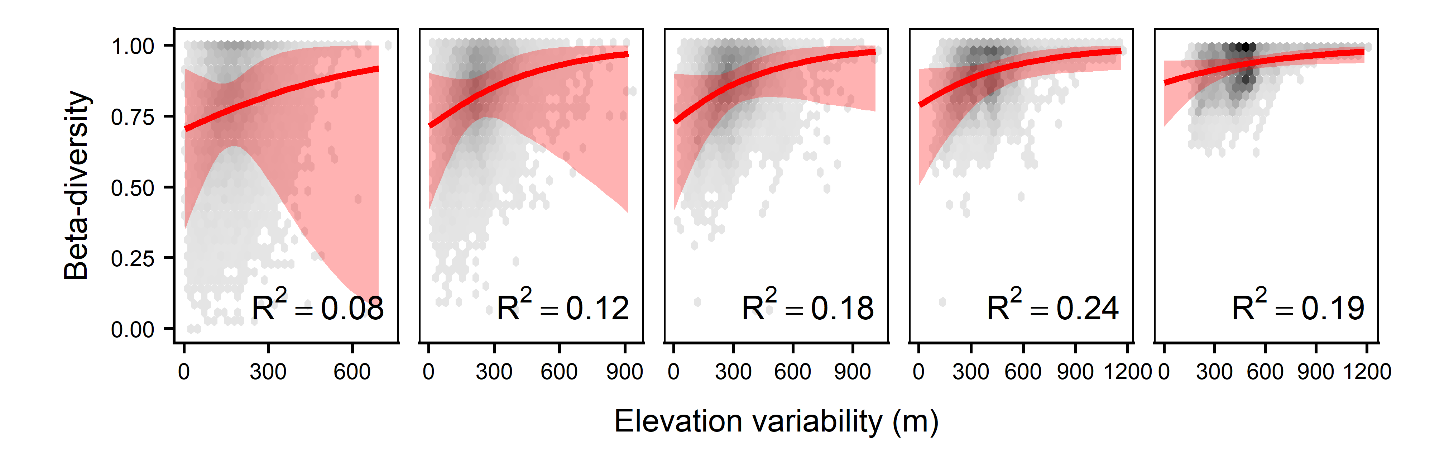
Supplemental Figure B-4. Beta regressions of beta diversity versus the standard deviation of elevation. Density of points in the scatterplot is represented by shading of hexagonal areas to avoid overplotting. The dark red line is the median predicted value of regressions fit with 100,000 spatially stratified random subsamples of the full dataset, each with approximately n=20. The shaded red area is bounded by the 2.5% and 97.5% percentiles of the predicted values from the regressions. The median R^2^ value of the regressions is shown in each panel.


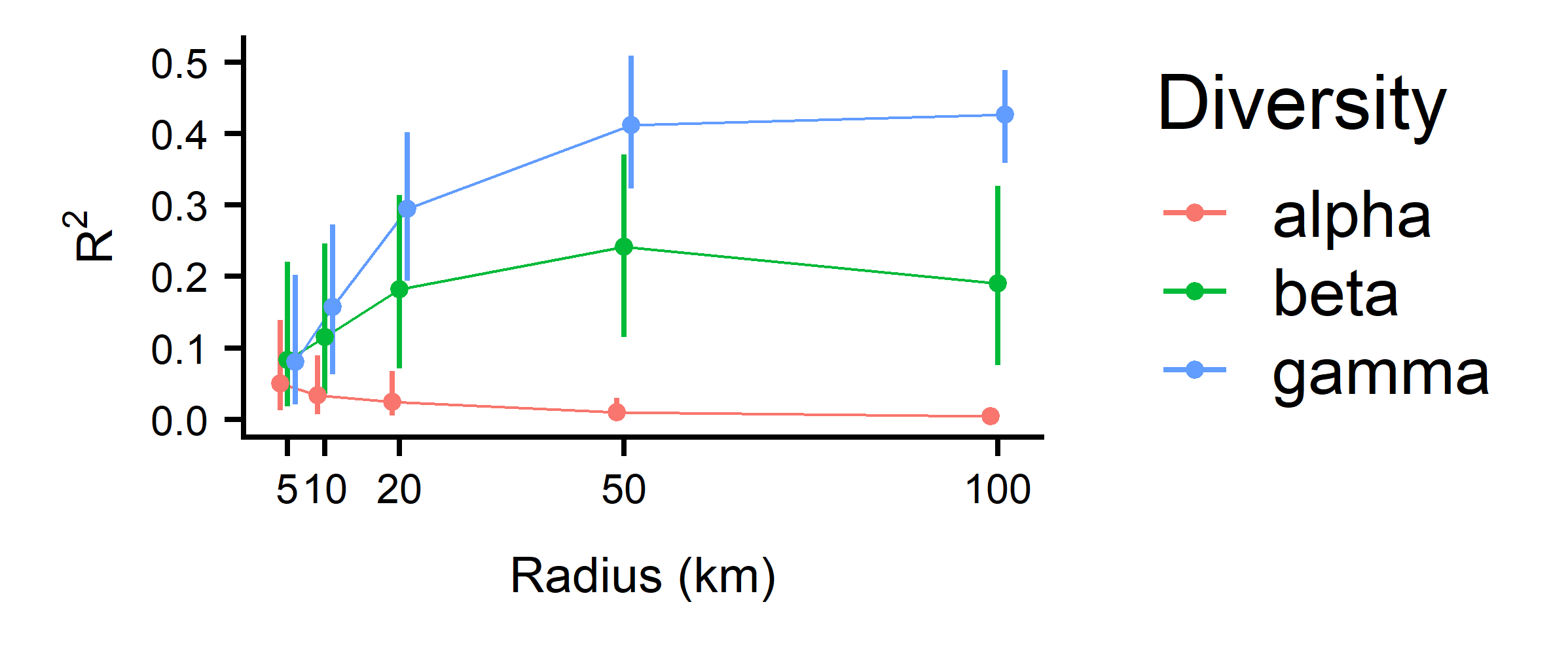


Supplemental Figure B-5. R^2^ values from biodiversity-geodiversity relationships shown in Supplemental Fig. B-3 (alpha diversity), Supplemental Fig. B-4 (beta diversity), and Box Fig. 1c (gamma diversity) at increasing grain sizes (radii around focal plot). The x-axis is increasing radii distance (km; akin to grain size). Error bars represent 25th to 75th percentiles.
